# Suicide Risk among Immigrants and Ethnic Minorities: A Literature Overview

**DOI:** 10.3390/ijerph15071438

**Published:** 2018-07-08

**Authors:** Alberto Forte, Federico Trobia, Flavia Gualtieri, Dorian A. Lamis, Giuseppe Cardamone, Vincenzo Giallonardo, Andrea Fiorillo, Paolo Girardi, Maurizio Pompili

**Affiliations:** 1 Department of Neurosciences, Mental Health and Sensory Organs, Suicide Prevention Center, Sant’Andrea Hospital, Sapienza University of Rome, 00189 Rome, Italy; alberto.forte@yahoo.it (A.F.); paolo.girardi@uniroma1.it (P.G.); 2 Psychiatry Residency Training Program, Faculty of Medicine and Psychology, Sapienza University of Rome, 00189 Rome, Italy; trobiafed@gmail.com (F.T.); gualtieriflavia@gmail.com (F.G.); 3 Department of Psychiatry & Behavioral Sciences, Emory University School of Medicine, Atlanta, GA 30303, USA; dalamis@gmail.com; 4 Psychiatric Department, Azienda USL Toscana Sud-Est, 53100 Siena, Italy; giuseppe.cardamone@uslsudest.toscana.it; 5 Department of Psychiatry, University of Campania Luigi Vanvitelli, 80138 Naples, Italy; enzogiallo86@gmail.com (V.G.); andrea.fiorillo@unicampania.it (A.F.)

**Keywords:** immigrants, ethnic minorities, suicide, prevention

## Abstract

Recent studies have demonstrated that immigrants and ethnic minorities may be at higher risk of suicidal behaviour as compared to the general population. We conducted a literature search to identify studies in English from 1980 to 2017 related to suicide risk among immigrants and ethnic minorities. Six hundred and seventy-eight reports were screened, and 43 articles were included in the qualitative synthesis of the review. Some studies reported lower rates of suicide attempts, while other findings suggested higher rates of suicidal behaviour and deaths among immigrants as compared to the native population. Also, a positive correlation was found between suicidal behaviour and specific countries of origin. Non-European immigrant women were at the highest risk for suicide attempts, a group which included young women of South Asian and black African origin. Risk factors among migrants and ethnic minorities were found to be: language barriers, worrying about family back home, and separation from family. The lack of information on health care system, loss of status, loss of social network, and acculturation were identified as possible triggers for suicidal behaviour. Overall, results suggest that specific migrant populations and ethnic minorities present a higher risk of suicidal behaviour than native populations, as well as a higher risk of death by suicide.

## 1. Introduction

Recently, a growing need for a better understanding of mental health issues among immigrants and ethnic minorities has emerged worldwide [1,2,3,4,5]. Europe is now facing the largest migration since the Second World War, resulting in new research questions about the extent of the burden of mental health disorders in migrants [1]. Migrants often experience physical and emotional trauma, including being victims of torture. This may be related to their risk of several psychological problems, such as post-traumatic stress disorder (PTSD), mood and anxiety disorders, and panic attacks, with symptoms of sleeplessness, nightmares, and flashbacks.

Several studies have suggested an increased risk of common mental disorders such as depression as well as psychotic disorders in immigrants as compared to native populations [6,7]. A meta-analysis suggested that migration could be considered a unique risk factor for severe mental health disorders such as schizophrenia [8]. Many studies also focused on women’s mental health, and suggested that immigrant women are at higher risk for postpartum depression than non-immigrant women [9].

Recent research has demonstrated that immigrants may be at a higher risk for suicidal behaviour [10,11]. Several authors have also suggested that suicide risk may vary among ethnic minorities [12], and they may have different and more specific risk factors for suicidal behaviour than the general population, such as acculturative stress [13]. As suggested by Wyatt and colleagues [13], acculturation (a process by which subjects acquire the attitudes, values, customs, beliefs, and behaviours of a different culture) may play a role in the development of a suicidal crisis among migrants and ethnic minorities, besides classical psychosocial and psychopathological risk and protective factors [13]. Moreover, the impact of acculturative stress on public health is complex and the underlying theoretical framework, its public health impact, and mental health implications are not yet clear [14,15]. To our knowledge, this is the first study providing a literature overview on suicidal behaviour and specific risk factors both in migrants and ethnic minorities. Thus, the aim of the present paper was to provide an overview of the literature on suicide risk among these two populations and explore potential differences between them.

## 2. Material and Methods

### 2.1. Search Strategy

In order to provide a novel and timely systematic review concerning suicidal behaviour among immigrants and ethnic minorities, we used the PRISMA (Preferred Reporting Items for Systematic Reviews and Meta-Analyses) statement for reporting systematic reviews [16]. We conducted a MedLine, Excerpta Medica, PsycLit, PsycInfo, and Index Medicus search to identify all papers and book chapters in English on the main topic for the period from January 1980 to March 2017. We used as specific key words: “suicide” (or suicidal or suicidality), “ethnic minorities” (or ethnicity, ethnic groups, ethnic communities), and “migrants” (or immigrants), including additional references with hand-searched bibliographies of screened articles.

### 2.2. Study Selection

**Figure 1:**
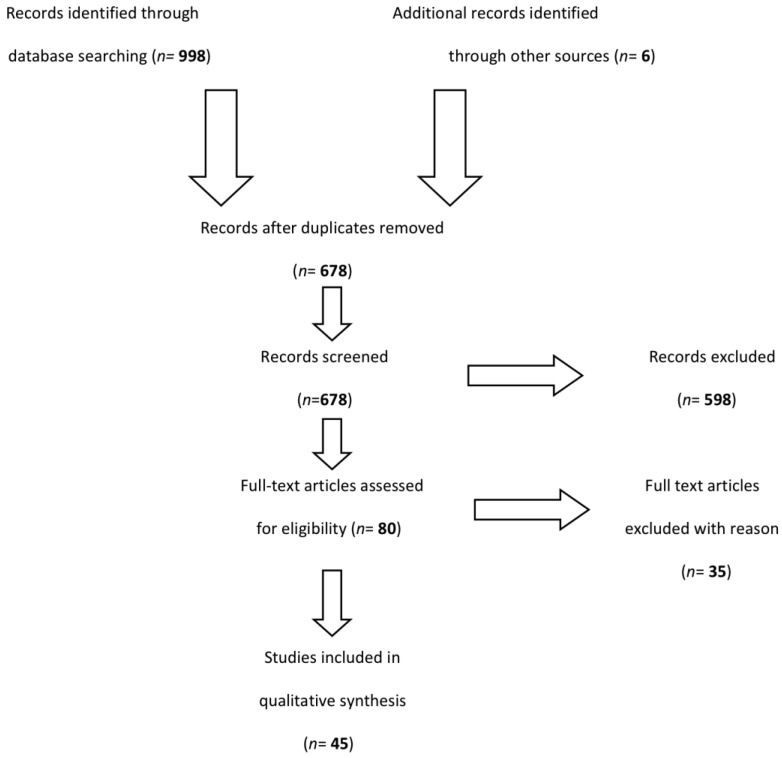
Search strategy and study selection.

As shown in Figure 1, a total of 998 abstracts were screened through the initial database searches. After duplicates were removed, a total of 678 reports were obtained and full texts were screened with a brief examination of titles and analysis of abstracts. Of these, 77 articles were identified as potentially relevant, and 45 were included in the qualitative synthesis based on the consensus of at least two authors. We gathered data from the initial 77 articles using a template form in Microsoft Excel, according to study characteristics, geographical area of interest, and results.

### 2.3. Eligibility Criteria

Articles that met the following criteria were included: (1) publication in a peer-reviewed journal between 1980 and 2017; (2) publication in English; (3) original study (e.g., not a review); (4) focus on suicidal behaviour; and (5) analysis on immigrants and/or ethnic minority population. The exclusion criteria applied were: failure to report on suicidal behaviour, sampling only from geriatric or paediatric subjects, repeated reports from the same study, and not reporting in English. Thirty-three articles did not fulfil the inclusion criteria, and the remaining 44 were included in the final review.

### 2.4. Study Concept, Quality Assessment and Terminology

The main aim of this research was to examine suicidal behaviour in immigrants and ethnic minority populations, reviewing the recent literature on this topic in order to identify and understand possible risk factors for suicide and attempted suicide in the investigated population.

A quality assessment was performed as shown in Table 1, rating studies with the following criteria: (I) sample size (not reported 0 points, <1000 1 point, ≥1000 2 points, ≥10,000 3 points); (II) study method (1 or 2 points); (III) evidence-based measures assessing suicide or suicide attempts (0 or 1 points); and (IV) reliability of data (1 or 2 points). The maximum score obtainable for each study was 8.

To clarify the terminology used in the article, suicidal behaviours refers to both suicide (resulting in a fatal outcome), attempted suicide, and suicidal ideation (thoughts about suicide such as plans and ideas) [17].

With the term migrant (or immigrant), we refer to the definition of the International Organization for Migration (IOM). Although there is no formal legal definition of an international migrant, most experts agree with IOM, which describes a migrant as any person who is moving or has moved across an international border or within a state away from his/her habitual place of residence regardless of: (1) the person’s legal status; (2) whether the movement is voluntary or involuntary; (3) what the causes for the movement are; or (4) what the length of the stay is. In addition, the types of migration can be categorized into short-term migration (within 3 and 12 months), and long-term migration (for a duration of one year or more).

The term refugee refers to the definition of the Statute of the United Nations High Commissioner for Refugees (UNHCR), which delineates a crucial legal difference with respect to the migrant population [18]. Although it is common to see the two terms used incorrectly as synonyms, refugees are specifically all persons who are outside their country of origin for reasons of violence, persecution, or conflict. As a result, they require international protection and are covered by international law.

We found some discrepancies with regards to the use of the term ethnic minority. There is no legal definition of ethnic minorities in international law; however, in Europe the definition is provided by the European Charter for Regional or Minority Languages and by the Framework Convention for the Protection of National Minorities. National (ethnic) minorities can be theoretically (not legally) defined as a group of people living within a national state with the following characteristics: (1) status of a minority among the citizen of that state or representing a smaller group than the rest of the population of the state; (2) distinct culture, language, religion and will to preserve them; (3) not in a dominant position; and (4) long-term presence in the territory [19].

## 3. Results

### 3.1. Suicide Attempts and Ideation in Immigrants

As shown in Table 1, some studies reported that immigrants have lower rates of suicide attempts than the native population, with annual suicide mortality rates of 1.79 per 100,000 among adolescents with migrant status, compared to 3.05 for native-born individuals [20]. Moreover, a significantly decreased risk of depression was also reported: 10.8% for the migrant group, as compared to 18% for non-migrant group [21]. Conversely, other studies did not find a significant difference in rates among migrants and natives [22], or found mixed results [23,24,25,26]. Taken together, a majority of studies showed higher rates of suicide attempts among immigrants than the native population [27,28,29]. Several years ago, in Sweden, a higher risk ratio (1.3) was found for attempts in both men and women of foreign-born minorities as compared to the native population [30]. In England, suicide rates in migrants ranged from 15 to 17.4%, which are above the native-born rates [31,32]. Filipino migrants working in home care in Israel reported a prevalence of lifetime suicide attempts of 4.5% as compared to a rate of 1.4% in their home country [33]. Similar results were found among ethnic minorities in the Netherlands: Turkish and Surinamese females had a higher risk of attempted suicide, with a rate of 483/100,000 compared to 246/100,000 of Dutch females of the same age [34]. Duldulao and colleagues discovered that first-generation United States-born Asian American women had a significantly higher prevalence of suicidal ideation than their other national counterparts [35]. Overall, data suggested that non-European immigrant women are at the highest risk of suicide attempts [23,32,34], although a positive correlation between suicidal crisis and specific countries of origin was found. A rate of 37.7/1000/year was found for South Asian women as compared to 23.3/1000/year for white women and 23.9/1000/year for black women [36]. Black and Asian minority groups in Europe and the USA were shown to have an increased risk of developing mental health problems as well as a higher risk of associated factors for suicide attempts [23,36,37].

### 3.2. Suicide Deaths in Immigrants

Taken together, results indicated a higher risk of suicide deaths among immigrants and ethnic minorities. Shah and colleagues demonstrated that suicide mortality ratios in foreign males living in England and Wales were generally higher in those of younger age groups (between 20–24 and 50–54 years) born in Eastern European or Caribbean regions. The rates were generally lower in those born in Western Europe in the younger age group, and higher in the age bands of 75–79 and 80–84 years of age. Woman in older age bands (70–74 and 85+ years) and coming from African, Caribbean, and Chinese countries were found to have a higher suicide mortality ratio [38]. Bhui highlighted higher suicide rates among young women of South Asian origin and black African origin, with SMRs (Standardized Mortality Ratios) of 2.8 and 2.7, respectively [39].

### 3.3. Suicidal Ideation and Attempt among Ethnic Minorities

Studies have shown higher rates of suicidal behaviour and self-harm in ethnic minorities. O’Keefe and colleagues reported that American Indian suicidal ideation can be predicted by thwarted belongingness and perceived burdensomeness [40]. The study involved 171 American Indians (representative of 27 different tribes) and employed an online survey based on an empirically supported theoretical model of suicide—the Interpersonal-Psychological Theory of Suicide [72]. The results showed that the interaction of thwarted belongingness and perceived burdensomeness predicted suicidal ideation better than each predictor taken individually [40].

Walker and colleagues investigated the role of acculturation in suicidal-behaviour among African descendants living in the USA, in a sample of 423 adults using the *African American Acculturation Scale*. Contrary to previous studies, it was found that just religious well-being, and not acculturation, was predictive of suicidal ideation and history of suicide attempt [46].

Else and colleagues identified, among Hawaiian youth, high levels of acculturation as a risk factor for suicide attempts [73]. Moreover, Scheel examined suicidal behaviour among American Indian college students [41]. They found that American Indian college students’ suicidality was characterized by: a rate of suicidal ideation comparable to general college students and low rates of awareness of traditional tribal suicide (10%). Moreover, 57% of the participants with suicidal ideation reported that they would not seek help from a mental health professional. Furthermore, they identified that the help-seeking likelihood depended on the cultural commitment. Participants more committed to tribal culture prefer counselling from American Indian counsellors (e.g., tribal healer/medicine man: 22%; American Indian mental health professional from university: 24%; from the campus: 44%; outside the campus: 41%). Less committed participants instead showed a moderate openness to seeking help from non-American Indian counsellors [41].

### 3.4. Suicide Deaths in Ethnic Minorities

Studies have shown increasing rates of suicide deaths, especially in youths from ethnic minorities [42,73]. In Native Hawaiians, death by suicide is a relatively frequent phenomenon among adolescents and young adults, as they have higher lifetime prevalence rates of suicide attempts (12.9%) in comparison with non-Hawaiian students (9.6%) [73].

On the other hand, among the ethnic minorities living in the United Kingdom, black Caribbean (SMR = 0.26) and South Asian (SMR = 0.4) women showed a lower risk of suicide compared to the native population [44]. Black Caribbean and young Black African men (SMR = 2.05) were revealed to have, instead, a higher risk of suicide than the native population [44]. In Hawaiians and other Pacific Islander populations, rates and distribution of suicide deaths were similar to other indigenous population (such as Maori in New Zealand), with a first peak in adolescents and young adults (annual suicide mortality rates per 100,000 by age: 52.8/100,000 for 15–24-year-olds, increasing to 72.4/100,000 for 25–44-year-olds) and then a sharp decline instead of a second peak in the elderly, which is typical of the bimodal distribution in United States and most Western countries [73].

Wong and colleagues focused on youth risk factors for suicide in ethnic minorities among American high school students, using data from the 1999–2009 Youth Risk Behaviour Surveys [42]. Native Hawaiian/Pacific Islander adolescents and multiracial adolescents had a higher prevalence of risk factors for suicide (such as depression, suicide ideation, plans, attempts and/or severe attempts), comparable to that of American Indian/Alaska Native adolescents already known in the literature as an “at-risk population” [42].

Bhui and colleagues also investigated the possible role of ethnicity in suicidal behaviour and related risk indicators among patients within a year of contact with psychiatric services [39,44]. Although they revealed that ethnicity influenced suicide rates and indicators of suicide risk, classical indicators of suicide risk such as “risk-related symptoms” (suicidal ideas, depressive symptoms, emotional distress, and hopelessness) were less common in ethnic groups such as black Africans (SMR 2.05) and South Asians, as compared to white British individuals [44]. Another study conducted by Ngwena in London public health services focused on trends of suicidal behaviour in black and minority ethnic (BME) groups. Among 996 BME patients admitted to the acute psychiatric ward with suicide attempt or self-harm-injury, those of Arab origin and South Americans were more prevalent (28%), followed by Western and Eastern Europeans (26%) [45].

Hunt identified the characteristics of psychiatric patients from ethnic minorities who died by suicide through the observation of 282 suicidal patients from ethnic minorities (6% of total) who had been in contact with mental health services in the 12 months prior to death. In these patients, suicide was characterized by: more violent methods, first episode of self-harm, higher rates of schizophrenia, unemployment, history of violence, and drug misuse [43].

### 3.5. Cultural Stress as Risk Factor for Suicidal Behaviour in Immigrants and Ethnic Minorities

Hagaman and colleagues investigated suicides among Bhutanese refugees resettled in the United States between 2008 and 2011, attempting to identify psychological characteristics and risk factors. Considering the small sample size, they showed a large presence of different post-migration difficulties in the investigated population [48]. The most common risk factor was a language barrier (71%), which seems to contribute to the development of hopelessness. Separation from family (43%) and worrying about family back home (57%) were also recognized as contributing factors to suicidal behaviour. Ngwena suggested other risk factors for self-harm, suicide, or suicide attempt in black and minority ethnic groups: arranged/forced marriage, lack of information on the health care system, loss of status and loss of social network, and also acculturation and thwarted ambition [45]. High levels of acculturation was identified as a risk factor for Hawaiian youth suicide attempts in another study [73]. In African descendants living in the USA, religious well-being was found to be a predictor for suicidal ideation [46].

Wong confirmed that multiracial adolescents more frequently reported a liability to mental disorders and health risk behaviours or dangerous conducts, such as substance use and violence [42]. In addition, immigrant status contributed negatively to acculturative stress, cultural conflicts, and socioeconomic difficulties. In England and Wales, as compared to native white people, suicidal ideation, emotional distress, and hostility were found to be less frequent among black Africans, black Caribbean individuals, and South Asians, while depression and a sense of hopelessness were more prevalent among South Asians [39]. These differences between native people and minorities suggest potentially different pathways to suicidal behaviour among different populations and that cultural stressors play a key role as risk factors for suicidal behaviour.

## 4. Discussion

The present overview suggested that findings on suicidal behaviour in immigrants are complex and it is difficult to delineate a theoretical framework explaining findings from the literature. On one hand, several studies found higher rates of suicide attempts among immigrants compared to native populations and that immigrants have a higher risk of experiencing suicidal behaviour than the same population in their home countries [27,28,29,74]. On the other hand, some studies did not reveal differences in suicidal behaviour between immigrant and native populations [22]. However, among the immigrant group and ethnic minorities, specific populations should be considered with regard to their risk of attempting suicide and also for suicide deaths, such as South Asian and black African women [36,39]. In terms of the literature related to immigrants, we found a lack of studies related to suicidal behaviour in ethnic minorities. The highest risk of suicide was found among black Caribbean individuals and young black African men living in the United Kingdom, suggesting that this might be related to specific environmental and psychosocial risk factors [44]. Some authors focused specifically on Native Hawaiians [73] and American Indian/Alaska Native adolescents [42], which have been found to be populations at risk for suicide death.

Various studies have also highlighted that immigrants and ethnic minorities do not receive the same psychiatric care during or after a suicide attempt or are less likely to contact psychiatric services when experiencing suicidal thoughts and/or engaging in suicidal behaviour [33,39,43]. This may lead to a global worsening of their mental health condition and an increased risk of suicide. In addition, if we take into consideration the different rates of suicide attempts and deaths, evidence indicates various patterns and mechanisms.

According to the Interpersonal Psychological Theory of suicide, perceived burdensomeness is a key ingredient for a heightened risk of suicide, together with low belonging, social alienation and the ability to enact self-injury behaviours [72]. Although Joiner’s theory holds true for non-minorities and non-immigrants as well, it can provide an explanation of the underlying mechanism related to the increased risk of suicide presented by these populations. Indeed, socioeconomic factors such as poor socio-economic status, social exclusion, discrimination, or deprivation [45,75] are more clearly related to suicidal behaviour rather than the status of migrant itself [25,64]. In conclusion, our findings suggest that migrants and ethnic minorities may be considered as a moderate–high risk group for suicidal behaviour, but this requires further and more specific investigation. Interestingly, ethnicity is related to different risk factors for suicide that are common among general population, such as; suicidal ideas, depressive symptoms, emotional distress, and hopelessness. These risk factors are less common in ethnic groups such as black Africans and South Asians [44]. This suggests that ethnic minorities may manifest a specific phenotype of suicidal behaviour; however, more studies are needed to understand the underlying mechanisms that may explain these differences. Further studies are needed to develop specific suicide prevention strategies targeting ethnic minorities, which represent an effective public health intervention.

### Limitations

The present review should be considered in the light of several limitations. First, the articles included in our synthesis were significantly different in terms of sample size, study design, and methodology, and this made it difficult to provide a quantitative synthesis. In addition, the samples included in this review varied widely on age, instruction, culture, traditions, and religious beliefs. Future studies should focus on investigating different rates and patterns of suicidal behaviour in the same population of migrants or ethnic minority longitudinally, in order to evaluate the influence of integration and acculturation on reducing suicidal behaviour.

## 5. Conclusions

Taken together, a majority of studies demonstrated higher rates of suicide attempts among immigrants than the native population. Risk factors among migrants and ethnic minorities were found to be: language barriers, worrying about family back home, and separation from family. Lack of information on the health care system, loss of status, loss of social network, and acculturation were identified as possible triggers for suicidal behaviour. Both migrant populations and ethnic minorities may have unique risk factors for suicidal behaviour; however, more studies are needed to clarify them. Additional research is required in order to develop a more reliable and standardized assessment of suicidal behaviour in these populations and to identify dedicated strategies of risk prevention for each group. It is also relevant for future studies to address the specific factors that influence suicidal thoughts and behaviours in each different ethnic group.

## Figures and Tables

**Table 1:** Summary of reports on suicide risk in ethnic minorities and immigrants stratified by region.

Study	Quality Score	Country	Aim and Study Design	Sample	Attempt *N*	Suicide *N*	Methods	Main Findings
**Ethnic minorities**								
O’Keefe et al., 2014 [40]	I:1 II:1 III:0 IV:1 Total: 3	USA	Investigated American Indian students’ suicidal ideation and its predictability. Observational study.	171	not reported	not reported	On-line survey.	Thwarted belongingness and perceived burdensomeness predict suicidal ideation in American Indians.
Scheel et al., 2011 [41]	I:1 II:1 III:0 IV:1 Total: 3	USA	Examined American Indian college students’ suicidality. Cross-sectional survey.	272	not reported	not reported	Suicidal Risk Questionnaire (SRQ), Cultural commitment and demographic questionnaire, Suicide-related beliefs and help-seeking preferences questionnaire.	Suicidal ideation comparable with general college students. Low awareness of traditional tribal suicide (10%). Participants more committed to tribal culture prefer counselling from American Indian persons.
Wong et al., 2012 [42]	I:3 II:1 III:0 IV:2 Total: 6	USA	Investigated youth risk factors for suicide in ethnic minorities among American high school students. Cross-sectional survey	88,532	not reported	not reported	Data from the 1999–2009 Youth Risk Behaviour Surveys (YRBS), a national survey of high school students. Questionnaire.	Native Hawaiian/Pacific Islander adolescents and multiracial adolescents have a higher prevalence of risk factors for suicide.
Hunt et al., 2003 [43]	I:1 II:1 III:1 IV:2 Total: 5	UK	Investigated suicides in ethnic minorities within 12 months of contact with mental health services in England and Wales. Clinical survey	282	not reported	282	Data from the Office for National Statistics (ONS) and questionnaire.	Among patients from ethnic minorities who had been in contact with mental health services in the 12 months before death, suicide was characterised by: violent methods, first episode of self-harm, high rates of schizophrenia, unemployment, history of violence, and drug misuse
Bhui et al., 2012 [44]	I:2 II:1 III:1 IV:2 Total: 6	UK	Investigated the influence of ethnicity on suicide, and related risk indicators among suicidal patients in contact with psychiatric services. Retrospective.	1358	not reported	1358	Data from the United Kingdom’s Office for National Statistics between 1996 and 2001. Questionnaire.	Black African men have higher rates of suicide as compared with the white British group. Classical indicators of suicide risk are less common in black Africans and South Asians, as compared with the white British group.
Bhui et al., 2008 [39]	I:2 II:1 III:1 IV:2 Total: 6	UK	Investigated suicide rates, symptoms, and preventability of suicide among suicidal patients within 12 months of contact with mental health services. Retrospective.	8029	not reported	8029	Data from the United Kingdom’s Office for National Statistics.	Rates and SMRs varied across ethnic groups.
Ngwena, 2014 [45]	I:1 II:1 III:1 IV:2 Total: 5	UK	Investigated trends of suicides in black and minority ethnic (BME) groups. Retrospective.	192	not reported	192	Data from the Office of National Statistics (ONS) and Public Health Observatory Mortality files, over the periods 2009–2012 and 2010–2013.	Suicides among patients from black and minority ethnic (BME) groups most prevalent in those of Arab origin and North or South Americans (28%), followed by those of Western and Eastern European origin (26%).
Walker et al., 2005 [46]	I:1 II:1 III:0 IV:1 Total: 3	USA	Investigated the role of acculturation in suicidal-behaviour among African descendants living in the USA. Cross-sectional survey	423	-	not reported	African American Acculturation Scale (AAAS). The Multi-Dimensional Support Scale (MDSS). The Spiritual Well-Being Scale (SWBS)	Religious well being, and not acculturation, is predictive of suicidal ideation and history of suicide attempt.
**Immigrants**								
***Continent of Origin: Asia***					-			
Chung et al., 2015 [47]	I:1 II:1 III:1 IV:1 Total: 4	USA	Risks and protective factors among Asian immigrants who repeatedly attempt suicide. Mixed-method study: Study 1 = retrospective study and Study 2 = clinical survey.	Study 1 = 44 Study 2 = 12	≥2–5/persons	0	Retrospective study (*n* = 44: clinical records) and clinical survey (*n* = 12 semi-structured interviews).	Among Asian immigrants with repeated suicide attempts, risk factors are: hopelessness, social isolation, self-stigma, feelings of failure, and sense of rejection by own family. Protective factors: psychological well-being, feeling cared for and able to reciprocate care for others.
Bhugra et al., 2002 [36]	I:1 II:1 III:1 IV:2 Total: 5	UK	Collected information on inception rates of attempted suicides across all ethnic groups, in the UK. Cross-sectional survey.	434	65	0	Data from the 1991 census. Semi-structured interview (schedule assessing the attempt, culture identity schedule, life events, GHQ–28, and Clinical Interview Schedule–R).	South Asian women, especially those aged 18–24, have higher rates of attempted suicide, in association with high rates of cultural alienation and previous attempts.
Dai et al., 2015 [21]	I:2 II:1 III:1 IV:1 Total: 5	China	Explored psychological consequences of internal migration among young rural Chinese and the associations between migrant status, mental health, and suicidal behaviours. Cross-sectional survey.	1646	10	0	Structured interview. Questionnaire (psycho-QOL subscale of the World Health Organization’s QOL Questionnaire—Brief Version; CEDS).	Socio-demographic and clinical variables, and social support, not migrant status, were the central determinants of mental health among all participants. Compared to their rural-residing peers, migrant workers had a decreased risk for depression and comparable risk for poor psycho-QOL and one-year serious suicide ideation.
Ayalon, 2012 [33]	I:1 II:1 III:1 IV:1 Total: 4	Israel	Investigated suicidal and depressive symptoms along with exposure to abuse and perceived social support in Filipino home care workers in Israel. Cross-sectional survey.	178	8	-	Questionnaires (Paykel Suicide Scale; Patient Health Questionnaire-9).	The Filipino sample in Israel showed higher levels of suicide attempts compared to national statistics in the Philippines. Abuse within the home/work environment (35% of the sample) was predictive for depressive symptoms (3.4% depressed)
Hagaman et al., 2016 [48]	I:1 II:1 III:1 IV:1 Total: 4	USA	Explored suicides among Bhutanese refugees in the USA. Cross-sectional survey.	14	not reported	14	Psychological autopsies, Hopkins Symptom Checklist-25 (HSC).	Suicide among Bhutanese refugees is connected with experiences of family withdrawal, integration difficulties, and perceived lack of care.
Duldulao et al., 2009 [35]	I:2 II:2 III:1 IV:2 Total: 7	USA	Investigated suicidal behaviours among Asian Americans, focusing on the correlates of suicidal ideation, plan, and attempt with nativity and gender. Prospective study.	2095	52	-	Interviews. Data from the National Latino and Asian American Study (NLAAS).	U.S.-born Asian American women showed higher prevalence of suicidal ideation and suicide plan than U.S.-born Asian American men and immigrant Asian American men and women.
Wong et al., 2014 [37]	I:2 II:1 III:0 IV:2 Total: 5	USA	Explored the correlation between the proportion of life in the USA and suicide ideation among Asian Americans in order to address within-group ethnic variability. Cross-sectional survey.	1332	-		Data from the National Epidemiologic Survey of Alcohol and Related Conditions.	Asian Americans in the U.S. had higher rate of suicide ideation, with significant differences within ethnic groups: the highest rates were found in Korean Americans and the lowest in Indian Americans.
***Continent of Origin: The Americas***								
Eaton, 2011 [49]	I:2 II:1 III:1 IV: 2 Total: 6	USA	Investigated the associations between racial/ethnic variations and risk of suicidal behaviours among Hispanic/Latina female students. Cross-sectional survey.	6322	Total: 575 White 318 African american 101 Hispanic 161	-	Data from The 2007 national school-based Youth Risk Behaviour Survey (YRBS);	Hispanic/Latina female students had a higher prevalence of suicidal ideation (21.1%) and suicide attempts (14.0%) than white and African American students. The risk behaviours associated with suicidal ideation and suicide attempts were: injuries and violence; tobacco use; alcohol and drug use; sexual behaviours; perceived health status.
Peña et al., 2008 [50]	I:2 II:2 III:1 IV: 2 Total: 7	USA	Investigated the associations between immigration generation status and suicide attempts, substance use and depressive symptoms among Latino adolescents in the USA. Cross-sectional survey (prospective cohort study).	3135	I generation 53 II generation 106 Later generation 153	-	Data from The National Longitudinal Study of Adolescent Health (Add Health). Interviews.	Among Latino adolescents, immigrant generation status is determinant for suicide attempts. Second-generation U.S.-born Latino youths were 2.87 times more likely to have made suicide attempts as compared to foreign-born youth. Later generations of U.S.-born Latinos were 3.57 times more likely to have made suicide attempts as compared to first-generation Latino youths.
Hovey et al., 2003 [51]	I:1 II:2 III:0 IV: 1 Total: 4	USA	Investigated suicide risk factors and depressive symptoms among Mexican migrant women farmworkers in the U.S. Midwest. Prospective study.	20	-	-	Interview and questionnaires (Adult Self- Perception Scale, Family Assessment Device, Beck Hopelessness Scale, SAFE Scale, The Personal Resource Questionnaire, Personality Assessment Inventory, Suicidal Ideation and Interview Topics, Center for Epidemiologic Studies Depression Scale).	Migrant women farmworkers presented elevated levels of anxiety, depression, and suicidal ideation. High depression symptoms were associated with: family dysfunction, ejective social support, hopelessness, and acculturative stress. High suicidal ideation was associated with lower self-esteem, family dysfunction, less ejective social support, hopelessness, acculturative stress.
Borges et al., 2009 [52]	I:2 II:1 III:1 IV: 2 Total: 6	USA	Explored suicidal behaviour among Mexicans and Mexican Americans. Retrospective study.	*n*1 = 5782; *n*2 = 1284	-	1284	Data from the Mexican National Comorbidity Survey (MNCS) (2001–2002; *n*1 = 5782) and the Collaborative Psychiatric Epidemiology Surveys (CPES): (2001–2003; *n*2 = 1284).	Migration to the USA is a risk factor for suicidal behaviour among Mexican people. Risk for suicidal ideation: having a family member in the USA; having arrived in the USA before the age of 12, and being U.S.-born. Risk for suicide attempts: having a family member in the USA and being U.S.-born.
Sorenson et al., 1999 [53]	I:3 II:1 III:1 IV: 2 Total: 7	USA	Cross-sectional survey	38,166	-	1344	Data from California Master Mortality.	The differences in mortality between foreign- and U.S.-born persons: young immigrants have lower or similar risk of death and they are underrepresented in suicides and overrepresented in homicides compared with U.S.-born persons.
***Continent of Origin: Africa***								
Shoval et al., 2007 [22]	I:0 II:1 III:1 IV:2 Total: 4	Israel	Investigated suicide in Ethiopian immigrants.	-	-	25/100,000	Data from National epidemiological surveys by the Israeli Ministry of Health.	High rates of suicide among Ethiopian immigrants in Israel, significantly higher than other immigrant populations.
Walsh et al., 2012 [54]	I:1 II:1 III:0 IV:1 Total: 3	Israel	Investigated the relations between suicidal ideation and alcohol abuse with ethnic identity and parental support among Ethiopian adolescents in Israel. Cross-sectional survey.	200	-	-	Questionnaires.	Suicidal ideation correlates with ethnic identity, alcohol use and parental support. A strong positive ethnic identity plays a protective role against suicidal and risk behaviours. Among Ethiopian adolescents in Israel, Ethiopian identity correlates with lower levels of suicidal ideation and alcohol use.
***Continent of Origin: Europe***								
Aichberger et al., 2015 [55]	I:1 II:1 III:1 IV:1 Total: 4	Germany	Investigated rates, motives and effectiveness of intervention-programs for suicidal behaviour among Turkish women in Berlin who made suicide attempts. Cross-sectional survey.	159	159	-	Questionnaires.	The high rate of suicide attempts among second-generation Turkish women in Berlin showed a significant reduction due to the application of the intervention program.
Ryan et al., 2006 [31]	I:1 II:2 III:0 IV:1 Total: 4	UK	Explored depression and suicide rates in Irish migrants in London. Case–control study	360	-	-	Questionnaire (Beck Depression Inventory (BDI).	Irish migrants in London show higher rates of depression and suicide compared with other minority ethnic groups, especially when associated with poorly planned migration. This effect can be modified by positive post-migration experiences.
Shah et al. A, 2011 [38]	I:0 II:1 III:0 IV:2 Total: 3	UK	Examined suicide rates in England and Wales and its correlation with ethnicity. Retrospective.	-	-	-	Data from the Office of National Statistics.	Differences in suicide rates among ethnic groups: male suicide rates were higher in all ethnic groups, except in the Chinese group, and increased with ageing among Indians; female suicide rates were higher among Chinese and increased with ageing among the African and Chinese groups.
Voracek et al., 2009 [56]	I:3 II:1 III:1 IV:2 Total: 7	Austria	Investigated the role of genetic risk factors for suicide, comparing immigrant suicide rates in Austria with country-of-birth ones. Retrospective.	65,206	-	65,206	Data from Statistics Austria.	Immigrant and homeland suicide rates were significantly positively associated. This evidence confirms the existence of genetic risk factors for suicide specific for each population.
Termorshuizen et al., 2012 [24]	I:3 II:1 III:1 IV:2 Total: 7	Netherlands	Examined ethnic density and suicide risk among migrant groups in four big cities in the Netherlands. Retrospective cohort study.	2,874,464	-	2572	Data from Statistics Netherlands.	The ethnic density influenced suicide risk among ethnic groups. The presence of their own ethnic group in the neighbourhood has a positive effect on suicide risk among non-Western minorities.
Di Thiene et al., 2015 [25]	I:3 II:2 III:1 IV:2 Total: 8	Sweden	Investigated differences in suicide between first- and second-generation immigrants in Sweden. Prospective population-based cohort study.	4,034,728	-	4358	Data from Statistics Swede: theNational Board of Health and Welfare.	The risk of suicide is lower in the first generation and higher in the second generation of immigrants compared with natives in Sweden.
Johansonn, 1997 [57]	I:3 II:1 III:0 IV:2 Total: 6	Sweden	Analysed the influence of ethnicity, age, sex, marital status, and date of immigration on suicide rates among immigrants in Sweden. Retrospective.	6,725,274	-	-	Data from Central Cause of Death Register.	Risk factors for suicide among immigrants in Sweden: ethnicity, being unmarried, male sex, age 45–54 or 75 and older, being born in Eastern Europe, Finland, or in non-European countries, and having immigrated to Sweden in 1967 or earlier. Males born in Russia/Finland and females born in Hungary/ Russia/ Finland/ Poland showed the highest risk ratios for suicide and higher risks than in their countries of birth.
Stack, 1981 [58]	I:0 II:2 III:0 IV:2 Total: 4	USA	Examined the association between immigration and suicide. Systematic cross-national investigation.	-	-	-	Data from the World Bank.	The rate of immigration has effects on the incidence of suicide: 1% increase in immigration is associated with a 13% increase in the rate of suicide.
Burvill, 1998 [59]	I:3 II:2 III:0 IV:1 Total: 6	Australia	Investigate suicide rates in migrant groups to Australia from Britain, Ireland, and the rest of Europe for the years 1979–1990. Epidemiological study.	-	-	-	Data from the Australian Bureau of statics (ABS) and the World Health Organization Annual Statistics between 1979 and 1990.	Comparison between the Australian-born and migrants from 11 European countries showed increased rates in immigrants compared with those in their country of birth (COB), and a significant rank correlation of the immigrant rates with those in their COB.
Värnik et al., 2005 [60]	I:3 II:2 III:0 IV:1 Total: 6	Estonia	Investigated changes in suicide rates among Russians in Estonia before (1983–1990) and after (1991–1998) Estonian independence from the Soviet Union. Epidemiological study.	-	-	-	Data from the World Health Organization and the Estonian Statistical Office.	Significantly higher suicide rate in Estonian Russians after Estonian independence in 1991, as compared to that of Estonians and Russians in Russia. The loss of privileged status of Russians during the Soviet era led to increasing suicide rates.
Maynard et al., 2012 [61]	I:0 II:1 III:0 IV:2 Total: 3	UK	Analysed suicide in England and Wales among immigrants. Cross-sectional survey.			0	Data from The Office for National Statistics (ONS).	This study indicated declining trends of suicide rates for most migrant groups and for English and Welsh-born women, but adverse trends for some country of birth groups.
***Continent of Origin: Australia***								
Taylor et al., 1998 [62]	I:0 II:1 III:0 IV:2 Total: 3	Australia	Examined the differences in suicide by socio-economic status (SES) in urban areas of Australia. Retrospective.	-	-	-	Data from the NSW unit record mortality tape, from the Australian Bureau of Statistics (ABS).	Risk of suicide in females was 28% that of males in adults, and 21% for youth. The risk increased significantly with decreasing socio-economic status in males, but not in females and depended on the country of origin.
Morrell et al., 1999 [63]	I:0 II:2 III:0 IV:2 Total: 4	Australia	Examined the differences in suicide rates between urban and rural areas of Australia among immigrants. Population-study.	-	-	-	Epidemiological data on the entire Australia population.	The higher rates of suicide in older males in non-metropolitan areas of Australia is mainly due to the high migrant suicide rates in these regions, but the same it is not true for the higher rates of male youth suicide in non-metropolitan areas.
Page et al., 2006 [64]	I:0 II:1 III:0 IV:2 Total: 3	Australia	Examined the correlation between trends in suicide and socio-economic status (SES) in Australia. Cross-sectional survey.	-	-	-	Data from the Australian Bureau of Statistics (ABS)	Socio economic status (SES) influenced suicide rates in Australia and the suicide rate was higher in low-SES males. The suicide rate was lower in young males in middle and high SES groups.
Law, 2014 [26]	I:2 II:1 III:1 IV:2 Total: 6	Australia	Investigated suicide rates in second-generation migrants in Australia. Cross-sectional survey.	5541	-	5541	Data obtained from the National Coroners’ Information System (NCIS).	Second-generation migrants in Australia had a lower suicide risk compared to first-generation migrants or locals (third-plus-generation), and this evidence could be explained by their better socioeconomic status.
Ide et al., 2012 [65]	I:3 II:2 III:0 IV:2 Total: 7	Australia	Investigated suicide of first-generation immigrants in Australia between 1974 and 2006. Epidemiological study.	-	-	-	Data from Australian Institute of Health and Welfare (AIHW).	First-generation male migrants in Australia had suicide rates that correlated to those of their countries of birth (COB), but not females. Rates are probably influenced by cultures, traditions, ethnicities, and the genetic predispositions of their home country. All COB groups showed suicide rates decreasing in time.
Lipsicas et al., 2012 [66]	I:3 II:1 III:0 IV:2 Total: 6	EU	Investigated suicide among immigrants in European countries. Cross-sectional survey.	27,048	-	-	Data from the WHO/EURO Multicentre Study on Suicidal Behaviour	Immigrants in European countries showed significantly higher suicide-attempt rates (SARs) than their hosts and had similar rates across different European countries as well as country-of-origin suicide rate.
Loh et al., 2007 [67]	I:1 II:1 III:1 IV:2 Total: 6	Singapore	Analysed suicide in Singapore. Cross-sectional survey.	640	-	640	Data from the Center for Forensic Medicine database.	The suicide patterns in Singapore showed a few changes with the passing of time. The characteristics that remained constant for suicides were: low prevalence for teenagers, higher prevalence for older adults, male/female ratio, and method of jumping from heights. Factors with increased prevalence were: unemployment and history of psychiatric disorder.
Lester, 1995 [68]	I:0 II:1 III:0 IV:2 Total: 3	USA	Retrospective.				Data obtained from Kramer et al. Predictor variables obtained from the Census Bureau.	The suicide rate of those born in non-contiguous states of America and abroad is predicted by social characteristics.
Kandrychyn, 2004 [69]	I:0 II:1 III:0 IV:2 Total: 3	Russia	Investigated how suicide rates vary in each geographic area due to social factors, migration, and ethnicity. Retrospective.	-	-	-	Data from the publication of Goskomstat—the Russian State Statistics Committee.	The suicide rates vary through Russian Federation and are higher in the north because of the historical prevalence of the Finno-Ugrian component in the north of the country.
Greenfield, 2006 [70]	I:1 II:2 III:1 IV:1 Total: 5	USA	Examine suicide among North American adolescent immigrant population. Prospective.	344	344	-	Questionnaire	Canadian immigrant adolescents presented lower suicide rates compared to non-migrant ones, and this is due to a lower rate of reported drug use among immigrants.
Kwan et al., 2007 [20]	I:2 II:1 III:0 IV:2 Total: 5	China	Examined suicide among Hong Kong adolescents. Cross-sectional survey.	4540	-	-	Questionnaire. Data from Census & Statistics Department.	This study indicated that suicide rates among immigrant adolescents in Hong Kong depend on duration of residence: short-duration (<10 years) correlated with lower suicide rates, and long-duration (>10 years or more) with higher suicide rates than the local-born counterparts.
Malenfant et al., 2004 [71]	I:0 II:1 III:0 IV:2 Total: 3	Canada	Examined suicide among Canadian immigrants. Cross-sectional survey.	-	-	-	Data from the Canadian Vital Statistics Data Base and the World Health Statistics Annual of the World Health Organization.	This study examined differences in suicide rates between immigrants and non-immigrant Canadian residents. Immigrants had 50% lower suicide rates, which increased with age and involved predominately males.
